# Effect of genotype and outdoor enrichment on productive performance and meat quality of slow growing chickens

**DOI:** 10.1016/j.psj.2024.104131

**Published:** 2024-07-29

**Authors:** Simona Mattioli, Elisa Angelucci, Cesare Castellini, Alice Cartoni Mancinelli, Wei Chenggang, Francesca Di Federico, Diletta Chiattelli, Alessandro Dal Bosco

**Affiliations:** Department of Agricultural, Environmental and Food Science, University of Perugia, Perugia 06124, Italy

**Keywords:** chicken, slow growing genotype, range enrichment, performance, meat quality

## Abstract

The optimization of animal welfare, meat quality, environmental impact, and economic sustainability in alternative poultry farming can be achieved by modulating several productive factors and improving the synergy between the chicken genotype and the outdoor environment. The objective of the study was to characterize 4 slow-growing chicken genotypes reared in free range conditions. Eight hundred chickens (SGs; 25 chickens/replicates/genotype/enrichment) belonging to the following genotypes, Red JA57 (**RJ**), Naked Neck (**NN**), Lohmann Dual meat-type (**LD**), and an Italian crossbreed (Robusta Maculata x Sasso, **CB**). were utilised and slaughtered at 81 d: The grazing areas were alternatively provided with enrichment constituted by strips of sorghum plants (**ENR**) or only grass (**NO ENR**). Productive performance (daily weight gain, daily feed intake, feed conversion ratio, live weight) were recorded weekly. Behaviour observations (walking and grass pecking), carcass and meat quality of breast and drumstick were also assessed in 15 chickens/replicate/genotypes/enrichment. Results demonstrated that both LD and CB showed the highest walking activity, but the different strains were differently capable of using the foraging resources (eating grass). The better productive performance was recorded in RJ followed by NN, CB and LD. In LD and CB, the different walking activities also affected the physico-chemical profiles (lower pHu, WHC, and lipids) of the breast and drumstick. The oxidative status was worse in CB than in the other groups (lower tocols, higher carbonyls), in both meat cuts. Fatty acid profile was also related to the genetic strain: a higher amount of n-3 polyunsaturated fatty acids was recorded both in the breast and drumstick of RJ and NN. The Healthy Fatty Index resulted excellent in all the chicken genotypes. In conclusion, the environment/animal interaction resulted as an important factor affecting the adaptability of genotypes to an extensive rearing system. All four genotypes, to different extents, showed good adaptability and production performance, with the exception of LD and CB, which were too light for the commercial supply chain requirements.

## INTRODUCTION

Good equilibrium between animal welfare, product quality, environmental impact and economic sustainability in alternative poultry farming (**APF** - free range, organic) can be achieved by modulating several productive factors including the synergy between the chicken genotype and the “natural” environment (Dal Bosco et al., 2021). From the first attempts to outline extensive rearing systems (Castellini et al., 2002), using fast-growing strains, the genetic companies and the poultry chains have progressively selected less productive strains more adapted to the outdoor systems (Cartoni Mancinelli et al., 2021; Mattioli et al., 2022b). Accordingly, the fast-growing genotypes, widely used in conventional production, present severe disadvantages when used in extensive systems (Guarino Amato and Castellini, 2022; Bonnefous et al., 2023b). Many efforts have been made to define the most adapted genotype to APF but there is still a scientific and technical debate on what criteria to use to classify chicken strain (Cartoni Mancinelli et al., 2021; Dal Bosco et al., 2021). In particular, this adaptability is often referred to the daily weight gain (**DWG**), even if in these systems, the presence of outdoor runs requires other specific characteristics (e.g., walking activity, thermotolerance, disease resistance; Guarino Amato and Castellini, 2022). Indeed, although DWG is considered a preliminary indication of adaptability, other traits should be considered. Cartoni Mancinelli et al. (2021) defined an adaptability score using a multifactorial approach to simultaneously consider several variables such as behaviors, body conditions and injuries in Slow Growing (**SG**) strains. This study underlined that DWG and genotype are strictly linked, but genotypes, independently on DWG, intrinsically affect adaptability to APF.

Moreover, in the case of organic farming, the compulsory rules imposed by the regulations in term of feed formulation do not always permit the respect of the requirements of modern chicken strains (high energy, protein, vitamins, and amino acids balancing), thus fewer demanding birds should be preferred. Considering the importance of the genotype selection (slow/medium growing, dual-purpose, crossbreed with autochthonous breeds, etc.) for APF, the present study aims to provide a further contribution on the interaction between four slow-growing genotypes and 2 different environmental situations on performance and meat quality of chickens.

A further element which affects the adaptability of chicken is related to the fact that a chicken strain, although adaptable to an outdoor environment, must necessarily feel safe moving within the grazing area. Different enrichments can render the outdoor more attractive offering different types of refuge, considering that the wild ancestors of chickens were attacked by birds of prey and modern poultry breeds still instinctively recognize the danger (Dal Bosco et al., 2016). The presence of outdoor enrichments can help the chicken feel safe from predators and more protected from the sun and natural elements so they can venture further away from the huts and better explore the pasture with metabolic and qualitative consequences for animal welfare and meat quality (Dal Bosco et al., 2012; Bonnefous et al., 2022).

This study is part of a H2020 project called PPILOW, which aims to co-create innovations to improve the welfare for poultry and pigs reared in organic and low-input outdoor farming systems. To do this, the project uses a participatory approach, involving all actors of the production chain from farmers to consumers for proposing, studying, and implementing practical solutions for welfare improvement. Furthermore, and this is the scope of this study, the project aims to provide a combination of practical solutions for welfare improvement that can be applied on a pan European basis with specific adjustments depending on the target market (national legislation and consumer preferences).

## MATERIALS AND METHODS

### Animals and Housing

The experiment was carried out during the late spring season of 2021 at the commercial farm affiliated to University of Perugia (Italy). Chickens were reared according to EU Regulation 834/07, EU Regulation 889/2008, and the Italian directives (European Parliament and Council of the European Union, 2013) on animal welfare for experimental and other scientific purposes. The experimental protocol was approved by the Ethical Committee of the University of Perugia (ID number: 62705 of 07/15/2020).

**Figure 1:**
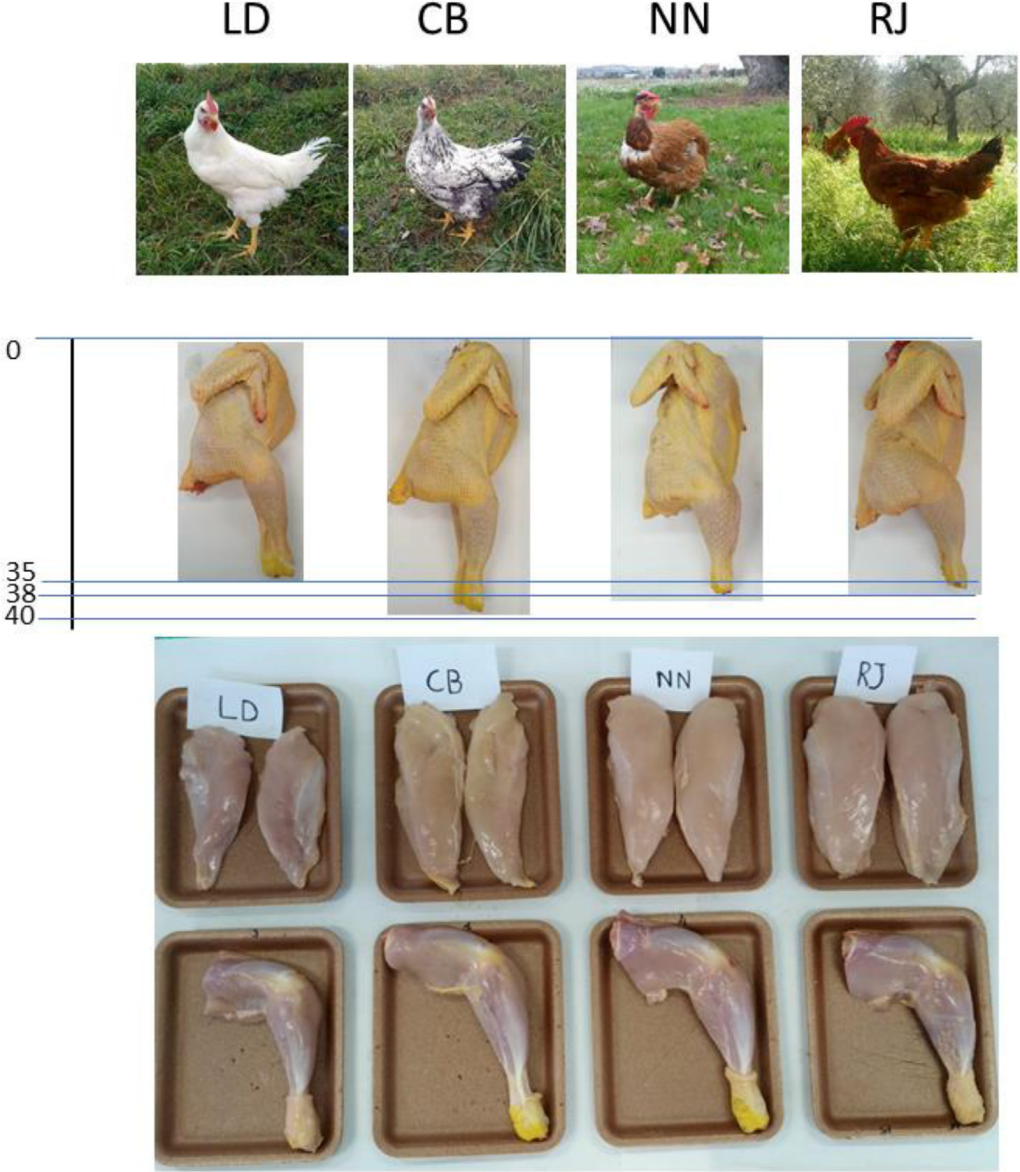
Pictures, carcasses (with cm scale; 0–40 cm) and meat cuts (breast, thigh and drumstick) of 4 chicken genotypes reared in an outdoor system with (ENR) or without (NO ENR) pasture availability.

A total of 800 chickens of four slow-growing genotypes (SGs; 25 chickens x 4 replicates x 4 genotype x 2 enrichment) were used. Specifically, the used genotypes were: Red JA57 (**RJ**), Naked Neck (**NN**), Lohmann Dual meat-type (**LD**) and an Italian crossbreed (Robusta Maculata x Sasso, **CB**; Figure 1). The birds were provided by 3 commercial poultry farms: RJ by Aviagen (Cocconato, AT, Italy), NN by Hubbard (Le Foeil-Quintin, France), LD by Lohmann breeders (Avizoo Lohmann Italy) and CB was produced in the experimental farm of the Department of Agricultural, Environmental and Food Science of University of Perugia.

Two replications of each chicken strain were reared in different pens that provided 128 m^2^ of outdoor space enriched or not with sorghum (total pen dimensions: 32 × 32 m) and which were also equipped with a shelter for the night. The indoor (0.10 m^2^/bird) and outdoor (4 m^2^/bird) densities of animals were specified according to organic regulations (EC Regulation no. 834/2007 and 889/2008). From 1 to 20 d of age, birds were housed in an environmentally controlled poultry house, with a temperature range 30 to 32°C and relative humidity 65 to 70% (monitored 24 h). At 21 days of age, the chickens were provided with free access to the outdoor space. The temperature and humidity of the outdoor were 19 ± 7°C and 50.1 ± 12.5%, respectively. The pasture was not treated with pesticides. The animals were fed *ad libitum* with the same diet (starter feed for 1–21 d, grower feed from 22 d to slaughter; Table 1); the diets provided chicken nutritional requirements as recommended by the breeding companies (HUBBARD BREEDERS https://www.hubbardbreeders.com/documentation/recherchedocumentheque.html). Water and feed were always available, and the birds were kept in shelters only during the night to protect them from predators.

**Table 1:** Dietary ingredients, proximate composition (% of dry matter, **D.M.**), energy value and nutrients of feed and grass.

		Starter	Finisher	Grass
*Ingredients*				
Maize	%	53.92	53.11	
Soybean meal	“	30.23	15.69	
Wheat	“	5.00	15.00	
Maize meal	“	5.08	11.45	
Gluten feed	“	1.00		
Soybean oil	“	0.62	1.15	
Vitamin-mineral premix1	“	0.40	0.40	
Dicalcium phosphate	“	1.71	1.21	
Calcium carbonate	“	1.23	1.29	
NaCl	“	0.20	0.23	
Sodium bicarbonate	“	0.15	0.15	
*Proximate composition*				
Moisture	%	12.20	12.00	78.61
Crude protein	% of D.M.	24.01	18.41	8.34
Ether extract	“	3.99	4.55	2.11
Ash	“	6.92	5.78	7.85
Crude fibre	“	3.48	3.60	23.2
NDF	“	17.63	10.1	60.90
ADF	“	7.41	5.06	39.81
ADL	“	1.67	1.11	5.81
Cellulose	“	5.74	3.56	34.0
Hemicellulose	“	10.22	5.05	21.09
Metabolizable energy^2^	kcal/kg	3245.20	3295.94	1876.00
*Nutrients*				
Vitamin A	mg/kg of D.M.	14.3	14.55	-
Vitamin E	“	67.5	55.03	355.51
Carotenes	“	2.16	3.65	401.65
C16:0	g/kg of D.M.	0.84	0.86	5.00
C16:1	“	0.01	0.01	0.21
C18:0	“	0.15	0.20	1.06
C18:1	“	1.60	1.65	7.53
C18:2	“	3.52	3.58	8.16
C18:3	“	0.27	0.29	8.56
SFA	“	0.99	1.06	6.05
MUFA	“	1.61	1.66	7.74
PUFA	“	3.79	3.87	16.72
n-6	“	3.52	3.58	8.16
n-3	“	0.27	0.29	8.56
n-6/n-3	-	13.04	12.34	0.95

^1^Amount per kg: vitamin A, 11,000 IU; vitamin D_3_, 2,000 IU; vitamin B_1_, 2.5 mg; vitamin B_2_, 4 mg; vitamin B_6_, 1.25 mg; vitamin B_12_, 0.01 mg; α-tocopheryl acetate, 30 mg; biotin, 0.06 mg; vitamin K, 2.5 mg; niacin, 15 mg; folic acid, 0.30 mg; pantothenic acid, 10 mg; choline chloride, 600 mg; manganese, 60 mg; iron, 50 mg; zinc, 15 mg; iodine, 0.5 mg; and cobalt, 0.5 mg.

^2^Estimated by Carré and Rozo (1990). SFA: saturated fatty acid, MUFA: monounsaturated fatty acid; PUFA: polyunsaturated fatty acid.

The grazing area consisted of 2 different typologies with (**ENR**) or without (**NO ENR**) outdoor enrichment constituted by four strips 2 meters wide and 20 meters long of sorghum (*Sorghum vulgare*) plants. An early sowing variety was chosen in such a way as to have, also thanks to irrigation, the sorghum plants already tall enough to provide a possible shelter for the chickens when the outdoor ranges in the ENR groups were opened. The height and density of the plants was lower than expected due the occurrence of late frost. The plant density was about 4 plants m^2^, 1.2 m high.

All the pens, regardless of the presence of the sorghum strips and considering the late spring season, were well grassed; the main species found in them were *Lolium perenne, Lotus corniculatus, Trifolium pratense, Phleum* spp., *Dactylis glomerata, Santolina* spp., *Agropyron* spp., *and Calamintha nepeta*. At the beginning of the experiment, one sample of grass per pen has been collected and transported to the laboratory for chemical analysis.

### Behavior Observations

Behavioral observations were performed in all pens using a computerized system (Noldus Technology, Wageningen, The Netherlands) consisting of 2 different software: Media Recorder to record the videos with the use of 8 cameras and Observer XT to analyze the videos. Cameras were positioned in advance on each pen to visualize all the space between the shelter and the distance of 5 m. From 42 to 81 d of age 2 videos/wk/pen of 2 h length (9.00–11.00 am) were recorded. Each video was analysed by 2 experts, observed by applying 10-min scan sampling interval using the reported ethogram (Table S1) (Cartoni Mancinelli et al., 2023). Data were expressed as the percentage of animals engaging in each behavior with respect to the number of visible animals/scan. The eating habit was estimated as the time spent by birds pecking grass/walking activity.

### Productive Performance and Carcass Traits

Once a week, 15 chickens/replicate from all genotypes were weighed to evaluate the daily weight gain (**DWG**, g/d/chicken). The feed consumption was recorded in every replicate by weighing the quantity of feed provided minus the feed that remained at the end of the week for measuring the daily feed intake (**DFI**, g/d/chicken). The feed-to-conversion ratio were also calculated (**FCR**).

At 81 d of age, all chickens were slaughtered in a commercial slaughterhouse 12 h after feed withdrawal; birds were electrically stunned (110 V; 350 Hz) before being killed and after bleeding, the carcasses were placed in hot water (56.5°C for 1 min) and then plucked and eviscerated (nonedible viscera, including intestines, proventriculus, gall bladder, spleen, esophagus, and full crop were removed), and the carcasses were stored for 24 h at 4°C. The following day, 32 chickens/genotype (8 chickens/replicate/genotype/enrichments) were selected and transported to the laboratory of the Department of Agricultural, Environmental and Food Science.

The ready to cook carcass was calculated, weighting the carcass after removing of neck, head, and legs. The bust and breast yield was also evaluated, such as cuts and stored fat weights, breast thickness, *sternum* and tibia lengths. The boneless drumstick meat and bone weights were also calculated, and the meat/bone ratio too. The breast and drumstick meat were excised from the carcasses, sampled and stored at -20°C for analytical evaluations.

### Proximate Composition and Technological Traits

The ultimate pH (24 h) was measured with a Knick digital pH meter (Broadly Corp., Santa Ana, CA) after homogenization of 1 g of raw muscle for 30 s in 10 mL of 5 M iodoacetate (Korkeala et al., 1986). The water-holding capacity (**WHC**) was estimated by placing 1 g of whole muscle on tissue paper inside a tube and centrifuging for 4 min at 1,500 x *g*. The water remaining after centrifugation was quantified by drying the samples at 70°C overnight. WHC was calculated as follows: (weight after centrifugation - weight after drying)/initial weight * 100. The drip loss was calculated evaluating the percentage loss in weight (starting from 30 g of meat) after dripping onto a metal grate for 24 h at 4°C. At 24 h postmortem, L* value (degree of lightness) was measured on the cut surface of meat using a tristimulus analyzer (Minolta Chroma meter CR-200, Osaka, Japan), following the CIELab color system (Robertson, 1977).

Moisture, ash, and total nitrogen were assessed using the AOAC methods (AOAC 1995 N. 950.46B, 920.153, and 928.08, respectively). Total protein was calculated by Kjeldahl using a 6.25 conversion factor. Total lipids were extracted in duplicate from 5 g of each homogenized sample and calculated gravimetrically (Folch et al.,1957).

### Analytical Determinations

*Antioxidants.* All oxidative parameters and fatty acid profiles were analyzed in duplicate. The α, γ−tocotrienols and α, γ and δ-tocopherols, and retinol contents of the meat were quantified using the HPLC system described below, according to Hewavitharana et al. (2004). Five milliliters of distilled water and 4 mL of ethanol were added to 2 g of sample and vortexed for 10 s. After mixing, 4 mL of hexane containing BHT (200 mg/L) was added, and the mixture was carefully shaken and centrifuged at 8,000 x *g* for 10 min. Three mL of the supernatant was dried under N_2_ and dissolved in 200 mL of acetonitrile; 50 mL was then injected into HPLC system (Hitachi Primade comprised of a cooling autosampler 1,210, pump 1,110, fluorimetric detector 1,440 and diode array detector 1,430 and a Synergi Hydro-RP column, Phenomenex, Bologna, Italy). The flow rate was 2 mL/min. All tocopherols and tocotrienols were identified using an FD detector (excitation and emission wavelengths of 295 and 328 nm, respectively) and quantified using external calibration curves prepared with increasing amounts of pure standard solutions (Sigma-Aldrich, Bornem, Belgium) in ethanol. The tocols sum was used for statistical analysis.

Retinol, considered as the final step of dietary carotenoids, was analyzed with the same HPLC system using a diode array detector set at λ 325 nm. Retinol was identified and quantified by comparing the sample with a pure commercial standard in chloroform (Sigma-Aldrich, Steinheim, Germany; Extrasynthese, Genay, France).

*Lipid Oxidation.* Lipid oxidation was evaluated using a spectrophotometer set at 532 nm (Shimadzu Corporation UV- 2550, Kyoto, Japan) that measured the absorbance of thiobarbituric acid reactive substance (**TBARS**) and a 1,1,3,3-tetraethoxypropane calibration curve (Ke et al., 1977). Oxidation products were quantified as malondialdehyde equivalents (µg MDA/g).

*Protein Oxidation*. Carbonyl derivatives of proteins were detected according to Mattioli et al. (2019). Briefly, the pellets from trichloroacetic acid (**TCA**) extracts were mixed with 1 mL of 10 mM DNPH in 2 M HCl. Samples were incubated for 1 h at RT and then centrifuged at 13,000 x *g* for 5 min. Supernatants were discarded and the pellets were washed 3 times with 1 mL of ethanol−ethylacetate (1:1, v/v) in order to remove unreacted DNPH. The pellets were then dissolved in 1.5 mL of 6 M guanidine-HCl and centrifuged as above to pellet insoluble particles. The carbonyl content of the resulting supernatants was evaluated spectrophotometrically (Shimadzu Corporation UV- 2550, Kyoto, Japan) at 370 nm using a molar extinction coefficient of 22,000 1/M*cm; values were expressed as nmol of carbonyl/mg of protein in the guanidine chloride solution. The same trichloroacetic acid extract was also used to evaluate thiol groups based on 5,5′ dithio-bis-2- dinitrobenzoic acid assay, with an extinction coefficient of 13,600 1/M*cm and expressed as µmol SH-group/g wet tissue.

With the aim to clarify the degree of cross-reactivity due to peroxide/aldehyde-protein interaction, which could lead to an underestimation of TBARS values, the lipid-protein cross-reactive level was reported as TBARS/Carbonyls ratio.

*Fatty Acids.* Total lipids were extracted from 10 g of each homogenized sample, according to Folch et al. (1957), and esterified according to Christie (1982). One ml of each solution containing fatty acid esters was transferred into vials for the gas-chromatographic analysis. The separation of fatty acid esters (**FAME**) was performed using a Varian gas-chromatograph (CP-3800) equipped with a flame ionization detector (**FID**) and an Agilent capillary column (100 m x 0.25 mm, CPS Analitica, Milan, Italy) coated with a DB-Wax stationary phase (film thickness of 0.25 µm). Injector and detector temperatures were set at 270°C and 300°C respectively. The carrier gas was helium at a flow rate of 0.6 mL/min. The oven temperature was programmed as follows: from 40°C (1-min hold) to 163°C (10-min hold) at 2°C/min ramp, to 180°C (7-min hold) at 1.5°C/min, to 187°C (2-min hold) at 2°C/min, and to 230°C (25-min hold) at 3°C/min. Single fatty acid methyl esters were identified by comparing their retention time with the retention time of commercially available FAME standard mixture (FAME mix Supelco 2560, Sigma-Aldrich, Germany). C21:0 methyl ester (CAS number 2363-71-5; Merck H5149, Germany), eluted under the same conditions of the samples, was used as internal standard (1 mg/100 µL of added solution). The area of each peak was used to calculate the fatty acid proportion. For the quantitative analysis (mg/100g of meat), the calculation method reported by Vahmani et al. (2017) was applied. Total saturated fatty acids (SFA), total monounsaturated fatty acids (MUFA), total polyunsaturated fatty acids (PUFA), total n-6 and n-3, as well as n-6/n-3 ratios, were also calculated.

Moreover, two indexes for measuring the desaturase ability of the different strains and the healthiness of the meat obtained have been calculated. The estimation of Δ5+Δ6-desaturase activity (Sirri et al., 2011) as following reported:

Δ5−desaturase+Δ6−desaturase=(C20:2n−6+C20:4n−6+C20:5n−3+C22:5n−3+C22:6n−3)/(C18:2n−6+C18:3n−3+C20:2n−6+C20:4n−6+C20:5n−3+C22:5n−3+C22:6n−3)

Healthy fatty acid index (HFI) was also calculated accordingly to Dal Bosco et al. (2022) applying the following equation:

$$\text{HFI}=[\left(\text{MUFA}\times 2\right)\pm \left(n-6\times 4\right)\pm \left(n-3\times 8\right)\pm^{n-3}{/}_{n-6}]/[\text{SFA}+\left(\text{MUFA}\times 0.5\right)+\left(n-6\times 0.25\right)+\left(n-3\times 0.125\right)+n-6/n-3]$$

All the values were expressed in mg/100 g of meat.

### Data and Statistical Analysis

Data obtained were summarized in the Table S2. All data obtained were statistically analyzed using a one-way ANOVA with the different genotypes, enrichment, and their interaction as fixed factors. Statistical significance was set at 0.05 and differences were assessed using Tukey's test. The SPSS (v18) statistical software was used.

## RESULTS AND DISCUSSION

The body of literature agrees that chicken genotypes with too high DWG and body weight are not adapted to extensive rearing systems because they have static behaviors, with a low use of outdoor range and generally exhibit a bad welfare status (Castellini et al., 2002). On the contrary, slow growing strains show more active behaviors and good welfare (Mattioli et al., 2022b). However, SG chickens with similar growth rate can show different adaptation to this rearing system due to intrinsic genetic characteristics of the strain (Cartoni Mancinelli et al., 2020).

### Behavioral Observations

**Figure 2:**
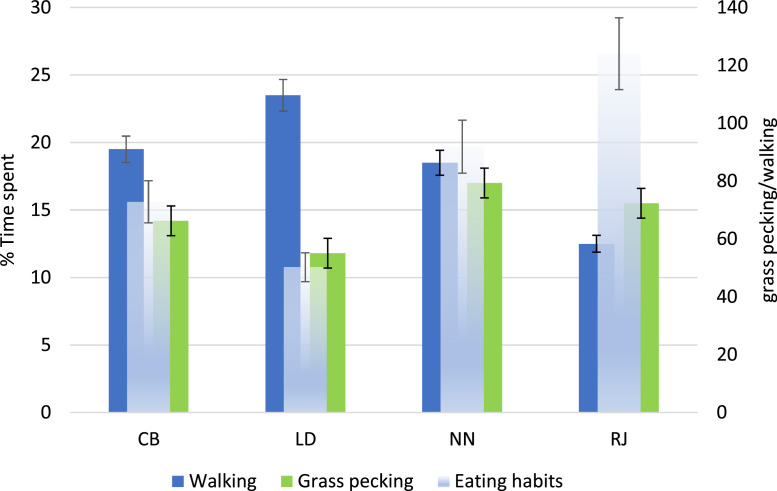
Time spent (%) in walking activity and grass pecking (eating behaviours was estimated as pecking grass/walking activity) of 4 slow growing chicken genotypes (95% lower and upper limits).

According to recent studies (Mancinelli et al., 2023; Collet et al., 2024) the explorative attitude of the chicken genotypes showed a high variability. The different genotypes showed a different walking and foraging behavior (Figure 2) without significant effect of the outdoor enrichment, although the presence of the sorghum strips tended to increase the movement in all the chicken strains (*p* > 0.05). The highest walking activity was showed by LD and CB, which were the less heavy chickens, confirming that the locomotory activity is affected by growth rate (Failla et al., 2021; Durosaro et al., 2023). The activity of pecking grass was generally connected with walking activity, a part in LD.

Other studies, (Mattioli et al., 2017; Bonnefous et al., 2022; Abdourhamane and Petek, 2024) showed that the foraging behavior of chickens is generally associated with their kinetic activity. However, as already shown in a preliminary experimental trial, which compared the same chicken strains, LD although having good kinetic activity did not also increase grass pecking. Indeed, LD showed the lowest ratio of walking/grass pecking (about 50.2) whereas RJ (124.0), followed by NN (91.9) the highest. This confirm that the DWG is not the only parameter affecting the adaptability to outdoor systems and that the intrinsic characteristic of the genotype, such as the behavioral component and explorative attitude, plays an important role (Cartoni Mancinelli et al., 2021). Part of this specie-specific behavior could be indirectly linked to the genetic selection done in meat-type strains. In fact, one of the primary responses to the increases in growth rate of chickens is the greater feed intake (Barbato, 1994). Accordingly, some SG meat-type commercial strains (e.g., RJ), which exhibited higher feed intake (Table 2) could exploit their kinetic activity in getting feed resources.

**Table 2:** Productive performance of 4 slow growing chicken genotypes reared with (ENR) or without (NO ENR) outdoor environmental enrichment.

		CB		LD		NN		RJ		RMSE	P value		
		ENR	NO ENR	ENR	NO ENR	ENR	NO ENR	ENR	NO ENR		GEN	ENR	INT
Chick weight	g	30.02	30.02	32.10	32.10	38.50	38.50	34.50	34.50	0.99	<0.001	0.729	0.803
Slaughtering weight	g	2,010.25	2,136.21	1,762.67	1,793.11	2,430.00	2,456.12	2,749.07	2,769.20	8.45	<0.001	0.089	0.075
DWG	g	24.45	26.00	21.37	21.74	29.52	29.85	33.51	33.76	0.88	<0.001	0.512	0.906
DFI	g	100.30	110.20	110.64	113.45	113.85	115.60	130.71	133.23	4.25	<0.001	0.099	0.075
FCR		4.10	4.24	5.18	5.22	3.86	3.87	3.90	3.94	0.15	<0.001	0.613	0.899

N= 4/genotypes; CB: crossbreed Robusta Maculata x Sasso, LD: Lohmann Dual, NN: Naked neck, RJ: Red JA57. GEN: Genotype. ENR: Enrichment. INT: Interaction GEN x ENR. DWG: daily weight gain; DFI: daily feed intake; FCR: feed to gain ratio.

At the same time, it should be underlined that the lack of effect of the environmental enrichment on the chicken behavior could be linked to the fact that, as previously mentioned in M&M, sorghum strips were not well developed due to the onset of late frost. Additionally, in a previous paper (Dal Bosco et al., 2014) we showed that the typology of enrichment affected the ranging behavior and the grass intake. Chickens reared under olive trees explored the available area up to almost 50 m from the hut, showing also a 20% more herbage ingestion, instead in a different outdoor enrichment (i.e., sorghum strips) they moved away by about half the distance. Thus, the characteristics of the outdoor enrichment should be further studied for having a more adapted conditions for the chickens.

### On Farm Productive Performance

Concerning the productive performance (Table 2), only the genotype has showed a significant effect on all the traits. The DWG of all the SG genotypes was either below 30 g/d or, in the case of RJ, only slightly above this level (∼33.6 g/d). RJ showed the highest slaughtering weight followed by NN and CB, whereas the LD had the lowest productive performance. Accordingly, RJ was characterized by the highest feed intake (130.70 and 133.23 g/d, respectively for ENR and NO ENR groups) whereas the better FCR was showed by NN (3.86 and 3.87, respectively for ENR and NO ENR groups), followed by RJ, CB and LD (5.18 and 5.22, respectively for ENR and NO ENR).

The value of FCR here obtained, in comparison to the conventional system, is relatively high and in line with what has been observed so far in SG genotypes reared free range (Cartoni Mancinelli et al., 2020, 2021). The high FCR was mainly due to the energy expenditure involved in non-productive activities (thermoregulation, walking, searching for food, specie-specific behavior, immune response– wing flapping; Bonnefous et al., 2023a). Even more, these strains are less precocious and require a longer time to reach the slaughtering weight. The combination of these two factors resulted in a decrease of feed efficiency due to the use of energy for purpose other than body growth.

As recently demonstrated in SG strains (Dal Bosco et al., 2021; Mattioli et al., 2022b), the ingestion of grass also dilutes the energy intake and the feed digestibility due to the increase of fiber (Tang et al., 2021; Luo et al., 2022). Sossidou et al. (2015) reported that the grass ingestion may limit the nutrient utilization of the diet and the feed efficiency. At the same time, Birolo et al. (2023) showed that an increase in dietary fiber, due to the partial substitution of traditional chicken ingredients (e.g., soya bean, corn) with local low input ingredients (e.g., fava bean), reduced the digestibility of protein and energy in all evaluated chicken strains, independently of their productive performance. In this respect, it should be underlined that the foraging activity in chickens has a low or no effect on ensuring macronutrients (e.g., energy, protein) for the growth, while a major effect on the bioactive content of the body and meat is generally observed (Dal Bosco et al., 2016).

### Postmortem Productive Performance

The carcass quality (Table 3) confirmed the same trend observed for the growth performance, with a lack of significance deriving from the outdoor enrichments, while the genotype had a significant effect for almost all the traits (apart the abdominal fat). CB and LD showed carcass traits (ready to cook carcass, breast characteristics, meat to bone ratio), not quite meeting the requirements of commercial distribution (Neeteson et al., 2023), whereas RR and NJ had a good carcass weight, a good carcass shape characterized by a high convexity, proportion of meat cuts (Figure 1) and meat bone ratio.

**Table 3:** Productive performance of 4 slow growing chicken genotypes reared with (ENR) or without (NO ENR) outdoor environmental enrichment.

		CB		LD		NN		RJ		RMSE	P value		
		ENR	NO ENR	ENR	NO ENR	ENR	NO ENR	ENR	NO ENR		GEN	ENR	INT
Ready to cook carcass weight	g	1,278.8	1,368.5	1,052.2	1,085.6	1,574.0	1,605.2	1,854.7	1,885.2	7.09	<0.001	0.729	0.803
Bust yield	%	63.6	64.1	59.7	60.5	64.8	65.4	67.5	68.1	1.22	<0.001	0.240	0.214
Breast weight	g	233.7	237.1	127.3	129.6	360.6	351.6	381.0	377.2	3.40	<0.001	0.939	0.972
Breast yield	%	11.08	11.24	7.20	7.60	14.84	14.47	13.33	13.33	0.58	<0.001	0.912	0.906
Breast thickness	cm	2.10	2.07	1.82	1.82	2.73	2.60	3.06	3.06	0.24	<0.001	0.613	0.899
Abdominal fat	g	1.58	1.68	2.35	2.40	2.16	2.50	2.55	2.60	0.55	0.480	0.060	0.278
Sternum length	cm	17.00	16.79	13.50	13.83	16.62	16.50	17.15	17.20	0.49	<0.001	0.077	0.044
Tibia length	cm	16.94	16.43	11.92	12.22	16.33	16.17	16.79	16.86	0.52	<0.001	0.546	0.736
Boneless drumstick weight	g	65.63	60.71	35.00	35.33	92.67	90.33	97.67	96.52	1.76	<0.001	0.607	0.957
Bone drumstick weight	g	37.50	37.86	19.33	19.67	48.00	44.50	48.67	48.00	1.28	<0.001	0.531	0.606
Meat/Bone ratio		1.75	1.60	1.81	1.80	1.93	2.02	2.01	2.01	0.16	0.069	0.647	0.572

CB: crossbreed Robusta Maculata x Sasso, LD: Lohmann Dual, NN: Naked neck, RJ: Red JA57. GEN: Genotype, ENR: Enrichment, INT: Interaction GEN x ENR.

### Breast and Drumstick Meat Physical Characteristics

Table 4 shows the physical characteristics of the breast and drumstick. Also, in this case, the genotype had a statistical significance, except for the brightness (L*) of both body cuts and the colorimetric coordinate b* of the drumstick. The colorimetric parameters of both the meat cuts showed great variability and trends that were difficult to interpret, however, CB showed a more intense colour (higher b*, a* and L) than NN and RJ, whereas LD showed intermediate values in the breast. This was not the same trend of the drumstick where higher a* and lower b* values were recorded in LD.

**Table 4:** Breast and drumstick meat physical characteristics of four slow growing chicken genotypes reared with (ENR) or without (NO ENR) outdoor environmental enrichment.

	CB		LD		NN		RJ		RMSE	P value		
	ENR	NO ENR	ENR	NO ENR	ENR	NO ENR	ENR	NO ENR		GEN	ENR	INT
*Breast*												
pH*u*	5.54	5.58	5.56	5.55	5.58	5.60	5.62	5.61	0.098	0.004	0. 081	0.204
L*	72.60	67.63	72.87	72.03	68.06	68.68	69.99	69.67	0.808	0.134	0.341	0.592
a	4.16	3.38	5.06	4.98	3.05	2.91	3.67	3.69	0.496	0.004	0.127	0.256
b	7.30	9.68	4.28	4.70	4.05	2.68	2.67	2.58	0.618	<0.001	0.383	0.184
Drip loss, %	0.45	0.45	0.55	0.56	0.40	0.46	0.45	0.44	0.583	<0.001	0.175	0.948
WHC, %	53.16	53.90	51.82	51.93	53.15	53.98	55.50	56.22	1.084	<0.001	0.617	0.960
*Drumstick*												
pH*u*	6.08	6.10	6.06	6.09	6.19	6.18	6.17	6.21	0.108	<0.001	0.940	0.333
L*	68.59	64.66	68.91	68.24	67.62	65.75	69.18	69.22	0.658	0.080	0.151	0.510
a	10.68	9.58	13.28	13.12	12.15	10.44	9.86	9.80	0.576	0.001	0.255	0.709
b	1.72	1.74	-0.14	0.32	1.44	1.33	1.93	1.58	0.549	0.118	0.885	0.986
Drip loss, %	0.74	0.74	0.86	0.85	0.27	0.32	0.23	0.22	0.716	<0.001	0.536	0.314
WHC, %	32.40	32.37	31.41	31.74	33.63	33.05	33.79	34.00	0.757	0.005	0.953	0.990

CB: crossbreed Robusta Maculata x Sasso, LD: Lohmann Dual, NN: Naked neck, RJ: Red JA57. GEN: Genotype, ENR: Enrichment, INT: Interaction GEN x ENR.WHC: water holding capacity.

On the contrary, differences in pH and WHC of genotypes were partially related to the walking activity and feeding behavior, which together with muscle metabolism, influenced the intramuscular fat content and the fatty acid composition. The higher motor activity of LD and CB strains probably determined a higher muscle glycogen store with a consequently lower pHu value, which led to a reduction of WHC, as also observed by Qiao et al. (2001). These differences in pH and WHC also led to significant differences in the Drip Loss of the meat which showed lower values negatively associated with the two previous parameters.

### Proximate Composition of Breast and Drumstick

In Table 5 the proximate composition of the breast and drumstick is reported. The different genotypes showed different lipid content in the breast and drumstick. As mentioned above, the genotype induced differences in the chemical composition of the breast and drumstick starting from the moisture content which, in the LD and CB, was lower than in the other strains. The different moisture observed also increase the protein content (*P* < 0.05 in drumstick) of chicken meat. Mueller et al. (2018), comparing different chicken genotypes, found a similar fat content in the leg of LD but a lower one in the breast (0.80 vs. 1.27 %). Dual purpose strains combine different genetic backgrounds (egg and meat-type parent) and this probably affected the allocation of dietary sources (Gous et al., 2018). Moreover, the higher fat content found in this study could be related to the use of a diet too concentrated in energy and protein for the specific requirements of LD strain. In fact, for experimental reasons, we used the same diet for all the genotypes, formulated based on more demanding strains (https://www.hubbardbreeders.com) and this amount of nutrients may be excessive for LD requirements.

**Table 5:** Breast and drumstick meat proximate composition of four slow growing chicken genotypes reared with (ENR) or without (NO ENR) outdoor environmental enrichment.

		CB		LD		NN		RJ			RMSE	*P* value		
		ENR	NO ENR	ENR	NO ENR	ENR	NO ENR		ENR	NO ENR		GEN	ENR	INT
*Breast*														
Moisture	%	74.82	75.71	74.33	74.85	77.89	76.82		77.00	76.71	0.88	0.009	0.837	0.966
CP	% F.M.	22.77	21.95	22.47	22.27	19.51	20.34		20.65	20.65	0.69	0.125	0.209	0.085
EE	“	1.01	1.07	1.27	1.28	1.46	1.44		1.63	1.71	0.16	<0.001	n.s.	<0.001
Ash	“	1.09	1.01	1.14	1.13	0.95	1.00		1.00	1.02	0.04	0.356	0.240	0.401
*Drumstick*														
Moisture	%	73.47	74.99	73.80	73.05	73.74	72.16		76.48	76.70	73.47	0.002	0.842	0.515
CP	% F.M	23.01	21.18	21.98	21.89	22.74	23.90		19.49	18.94	23.01	0.025	0.438	0.106
EE	“	2.41	2.74	3.00	3.28	2.34	2.57		3.08	3.22	2.41	<0.001	0.748	0.180
Ash	“	1.11	1.09	1.05	1.28	1.18	1.36		1.04	1.13	1.11	0.016	0.231	0.547

CP = crude protein; EE = ether extract; CB: crossbreed Robusta Maculata x Sasso, LD: Lohmann Dual, NN: Naked neck, RJ: Red JA57. GEN: Genotype, ENR: Enrichment, INT: Interaction GEN x ENR. CP: crude protein; EE ether extract.

### Oxidative Status of Breast and Drumstick

The antioxidant profile and the oxidative status of the meat are reported in Table 6. As generally established by many authors (Ponte et al., 2008; Fanatico et al., 2016; Dal Bosco et al., 2016, 2021; Mattioli et al., 2022b), movement and foraging aptitude of chickens in extensive poultry farming is positively correlated with the content of some antioxidants (tocols, carotenoids, polyphenols) in blood and muscle. Accordingly, APF positively affected the antioxidant content and the oxidative stability of the muscles. Probably, the small increases of foraging behavior in ENR groups, although not statistically evident (*p* > 0.05) from the behavior assessment, increased some antioxidants, thiols and reduced TBARS. Thiols come from grass, being an important class of phytochemicals (cysteine, γ-glutamylcysteine, glutathione, and phytochelatins) abundant in the vegetation (Qiang et al., 2005; Demirkol and Cagri-Mehmetoglu, 2008; Manda et al., 2010), which protect cells from oxidative damage.

**Table 6:** Breast and drumstick meat oxidative status of four slow growing chicken genotypes reared with (ENR) or without (NO ENR) outdoor environmental enrichment.

		CB			LD		NN		RJ		RMSE	*P* value		
		ENR	NO ENR	ENR		NO ENR	ENR	NO ENR	ENR	NO ENR		GEN	ENR	INT
*Breast*														
Retinol	µg/g	0.14	0.14	0.60		0.61	0.38	0.46	0.41	0.61	0.158	<0.001	0.089	0.334
γ-T3	µg/g	tr	tr	tr		tr	tr	tr	tr	tr	0.010	<0.001	0.541	0.759
α-T3	µg/g	0.21	0.24	0.07		0.05	0.11	0.07	0.07	0.04	0.121	0.001	0.649	0.887
δ-T	µg/g	0.31	0.28	0.11		0.11	0.15	0.13	0.16	0.12	0.096	<0.001	0.177	0.845
γ-T	µg/g	1.04	0.84	0.25		0.23	0.30	0.30	0.26	0.36	0.168	<0.001	0.068	0.036
α-T	µg/g	1.58	1.29	4.01		3.67	5.68	5.17	5.15	4.05	0.478	0.001	0.050	0.145
Σ Tocols	µg/g	3.14	2.65	4.44		4.06	6.24	5.67	5.64	4.57	0.486	0.018	0.050	0.177
Thiols	µmol SH-group/g wet tissue	3.47	3.06	4.10		2.99	3.60	2.97	4.12	2.98	0.365	0.589	0.014	0.100
TBARS	µg MDA/g	1.23	1.17	2.23		1.67	2.10	2.05	2.78	1.55	0.364	<0.001	0.051	0.213
Carbonyls	nmol/mg protein	2.73	3.99	1.09		2.27	1.11	0.62	1.13	1.11	0.383	<0.001	0.059	0.029
Lipid-protein cross reactive	-	2.22	3.41	0.49		1.36	0.53	0.30	0.41	0.72	0.258	<0.001	0.024	0.365
*Drumstick*														
Retinol	µg/g	0.32	0.29	0.77		0.73	0.61	0.58	0.55	0.64	0.171	<0.001	0.949	0.776
γ-T3	µg/g	0.01	0.02	tr		tr	tr	tr	tr	tr	0.070	0.008	0.062	0.062
α-T3	µg/g	0.58	0.61	0.04		0.07	0.13	0.09	0.06	0.03	0.149	<0.001	0.911	0.750
δ-T	µg/g	0.36	0.34	0.11		0.12	0.16	0.1	0.13	0.13	0.103	<0.001	0.328	0.199
γ-T	µg/g	1.98	1.97	0.37		0.32	0.45	0.3	0.66	0.48	0.244	<0.001	0.083	0.658
α-T	µg/g	3.36	2.05	6.08		5.15	9.34	5.52	7.29	6.11	0.607	<0.001	0.001	0.319
Σ Tocols	µg/g	6.28	4.97	6.60		5.66	10.08	6.01	8.14	6.75	0.619	0.001	0.001	0.029
Thiols	µmol SH-group/g wet tissue	3.16	3.47	4.05		3.06	3.62	3.15	4.14	2.28	0.402	0.864	0.023	0.147
TBARS	µg MDA/g	1.33	1.26	3.71		3.07	2.72	3.15	1.74	1.91	0.453	<0.001	0.992	0.716
Carbonyls	nmol/mg protein	6.54	7.37	3.81		1.25	2.87	3.15	2.39	2.07	0.555	<0.001	0.434	0.134
Lipid-protein cross reactive	-	4.92	5.85	1.03		0.41	1.06	1.00	1.37	1.08	0.365	<0.001	0.368	0.125

CB: crossbreed Robusta Maculata x Sasso. LD: Lohmann Dual. NN: Naked neck. RJ: Red JA57. GEN: Genotype. ENR: Enrichment. INT: Interaction GEN x ENR. Tr: trace i.e. <0.01 µg/g. T3: tocotrienol; T: tocopherol; TBARS: thiobarbituric reactive substances.

The oxidative status of the body (Fereidoon and Ying, 2010; Mattioli et al., 2022b) depends, a part the dietary intake of antioxidants, also from the interaction of the lipid profile of tissues with the oxidative stress produced by kinetic activity. The lipid profile affects the peroxidability of the system because high PUFA level, good for the nutritional profile of food, also reduces its stability (Shahidi and Zhong, 2010). The second one is related to the effect of locomotory activity on muscle metabolism and on the production of free radicals. In fact, the adaptation to exercise, is affected by the genetic strain (Mattioli et al., 2017) and could produce a completely different antioxidant status of the body. Accordingly, the results here obtained could be affected by the inputs entering in the chicken body but also by specific metabolisms, which for certain molecules (Perini et al., 2023) and processes, could be genetically driven (Mattioli et al., 2022a).

Thus, a general consensus between kinetic activity, eating behavior and antioxidant content is respected in RJ and NN, and the higher pecking grass activity improved the antioxidants and oxidative stability of muscle (Dal Bosco et al., 2016; Mattioli et al., 2021). As already discussed, LD birds showed the lowest grass pecking/walking ratio; however, the meat had α-tocopherol, total tocols and retinol higher than CB. It should be underlined that the antioxidants here analyzed, being lipophilic, are also affected by the fat content, which was very low in CB muscles. Indeed, fatter muscles could also contain higher retinol and tocols content. Notwithstanding this high antioxidant content, the drumstick of these chickens exhibited a higher oxidative process, probably due to the effect of movement on the muscles of the leg and to the amount of PUFA (Table 8).

Apart from α-T, which is the most abundant tocopherol in meat, it is interesting to underline that the content of δ- and γ- isoforms showed higher values in CB. The δ- and γ-isoforms are reported to be more effective like quencher against Reactive Oxygen Species (**ROS**) than α-T (Schubert et al., 2018). This trend suggests a particular metabolic pathway of CB compared to the other commercial genotypes also supported by the trends of lipid (**TBARS**) and protein (Carbonyls) oxidative processes. TBARS and Carbonyls are linked because the products of lipid oxidation (mainly peroxides and aldehydes) can promote protein oxidation in many food matrixes (Hematyar et al., 2019) by the way of formations of several adducts. In both the muscles of CB chickens extremely low quantities of malondialdehyde (low TBARS value) were observed. Then, it is plausible that to a lower TBARS value corresponds a higher carbonyl one due to a lipid-protein cross reaction. Accordingly, the discrepancies of CB trend should be read as the achievement of a state of lipid oxidation, which influences and triggers protein oxidation as well (see the value of lipid-protein cross reactive higher than TBARS).

The relationships between lipid (before) and protein oxidation (after) are extremely complex. Hellwig (2020) indicated that protein oxidation (protein carbonyl formation) occurs through multiple pathways, one of which is lipid dependent (as previously mentioned), and the other, lipid independent. As a result, the trend of carbonyls is more difficult to interpreter and did not seem affected by the presence or absence of environmental enrichment.

### Fatty Acid and Estimated Index of Breast and Drumstick

The FA profile and the calculated indexes (Δ5,6 desaturase, HFI) of both meat cuts are affected by genetic strain and environmental enrichments. In the ENR groups, as already occurred for the antioxidants, the fatty acid composition of breast and drumstick (Table 7, Table 8) showed a significant increase in linolenic acid (ALA, n-3) and docosahexaenoic acid (**DHA**) (only in breast) and a reduction, in the same muscle, of linoleic acid (LA, n-6) and arachidonic acid (**AA**).

**Table 7:** Fatty acids content (mg/100g of meat) of breast in four slow growing chicken genotypes reared with (ENR) or without (NO ENR) outdoor environmental enrichment.

	CB		LD		NN		RJ		RMSE	P value		
	ENR	NO ENR	ENR	NO ENR	ENR	NO ENR	ENR	NO ENR		GEN	ENR	INT
C14	2.67	2.99	3.50	3.30	2.96	4.05	4.61	5.84	0.41	<0.001	0.055	0.257
C16	187.63	201.94	220.35	230.38	265.60	329.39	358.51	361.99	2.64	<0.001	<0.001	0.001
C16:1	10.60	12.69	11.58	9.90	5.17	15.08	24.43	16.56	0.96	<0.001	0.712	<0.001
C17	1.88	4.92	3.28	3.76	4.43	2.61	3.80	2.81	0.53	0.990	0.766	0.054
C17:1	0.67	0.81	1.35	1.09	0.80	0.90	1.22	1.21	0.21	<0.001	0.940	0.375
C18	104.67	109.23	110.15	112.95	182.70	131.17	168.22	200.66	2.07	<0.001	0.587	<0.001
C18:1 n-9	211.03	241.75	244.03	218.74	247.16	252.13	367.73	342.78	2.81	<0.001	0.774	0.404
C18:2 n-6, LA	151.89	164.74	191.84	190.79	200.10	240.44	261.42	267.79	2.31	<0.001	0.020	0.023
C18:3 n-6, γ-ALA	0.58	0.69	2.53	2.19	1.05	1.03	1.03	1.08	0.44	0.013	0.897	0.977
C18:3 n-3, α-ALA	5.41	5.10	9.57	8.69	7.38	9.23	11.66	8.84	0.60	<0.001	0.950	0.009
CLAcis9trans11	1.05	1.09	0.33	0.17	0.43	1.91	0.23	0.37	0.31	<0.001	0.034	0.002
C20:2	2.83	3.04	3.28	3.72	3.87	3.66	4.39	5.41	0.35	<0.001	0.570	<0.001
C20:4 n-6, AA	80.60	78.90	70.25	79.34	108.20	111.72	107.21	124.00	2.18	<0.001	0.020	0.002
C22:2	1.21	1.13	0.62	0.64	1.08	1.97	1.54	1.75	0.24	<0.001	0.002	<0.001
C20:5 n-3, EPA	15.63	15.14	11.10	14.62	19.25	23.73	19.47	20.85	0.85	<0.001	0.125	0.377
C22:4	3.97	3.71	2.50	3.47	4.18	5.17	3.85	5.44	0.40	<0.001	0.008	0.273
C22:5 n-3, DPA	9.30	8.93	7.42	10.31	12.18	12.54	11.44	13.25	0.63	<0.001	0.008	0.039
C22:6 n-3, DHA	18.92	17.33	6.29	8.54	12.59	12.63	12.30	12.25	0.75	<0.001	0.223	0.205
SFA	296.85	319.08	337.28	350.39	455.70	467.23	535.14	571.30	3.23	<0.001	0.047	0.772
MUFA	222.31	255.25	256.97	229.73	253.14	268.12	393.38	360.54	2.94	<0.001	0.826	0.281
PUFA	289.71	297.27	307.31	323.86	369.85	421.18	431.80	460.99	3.02	<0.001	0.036	0.610
n-6	236.48	247.06	270.43	278.23	314.27	357.88	375.08	399.36	2.84	<0.001	0.046	0.663
n-3	53.23	50.21	36.88	45.63	55.58	63.30	56.72	61.63	1.15	<0.001	0.028	0.290
Long Chain PUFA	132.41	127.43	105.90	124.38	162.37	171.51	158.72	184.36	3.25	<0.001	0.058	0.290
n-6/n-3	4.44	4.92	7.33	6.10	5.65	5.65	6.61	6.48	0.33	<0.001	0.216	0.022
HFI	3.85	3.73	3.48	3.60	3.52	3.61	3.31	3.43	0.13	<0.001	0.329	0.499
Δ5,6 desaturase	45.70	42.87	34.46	38.41	42.90	40.72	37.76	38.99	1.25	<0.001	0.069	0.072

CB: crossbreed Robusta Maculata x Sasso. LD: Lohmann Dual. NN: Naked neck. RJ: Red JA57. GEN: Genotype. ENR: Enrichment. INT: Interaction GEN x ENR. LA: linoleic acid; α-ALA: α-linolenic acid; AA: arachidonic acid; EPA: eicosapentaenoic acid; DPA: docosapentaenoic acid; DHA: docosahexaenoic acid; SFA: saturated fatty acids; MUFA: monounsaturated fatty acids; PUFA: polyunsaturated fatty acids; HFI: Healthy Fatty Index.

**Table 8:** Fatty acids content (mg/100g of meat) of drumstick in four slow growing chicken genotypes reared with (ENR) or without (NO ENR) outdoor environmental enrichment.

	CB		LD		NN		RJ		RMSE	*P* value		
	ENR	NO ENR	ENR	NO ENR	ENR	NO ENR	ENR	NO ENR		GEN	ENR	INT
C14	11.56	12.49	9.39	11.07	9.74	10.00	15.49	15.79	0.55	<0.001	0.296	0.051
C16	450.93	589.37	388.31	524.49	397.4	458.94	535.96	576.53	3.36	0.150	0.213	0.022
C16:1	72.44	77.28	42.48	68.08	44.26	43.16	119.06	88.07	1.82	<0.001	0.793	0.000
C17	9.15	9.72	9.09	11.60	9.63	9.53	11.51	11.27	0.63	0.073	0.643	0.154
C17:1	4.11	4.59	5.03	5.76	5.07	4.98	4.92	6.54	0.45	0.009	0.187	0.056
C18	193.35	282.79	234.07	257.63	171.71	237.75	239.51	281.5	3.26	<0.001	0.578	0.001
C18:1 n-9	673.14	835.72	686.55	758.94	616.85	649.62	800.22	885.27	4.29	0.134	0.083	0.047
C18:2 n-6, LA	497.91	355.18	553.09	606.03	511.94	554.35	580.49	638.46	3.61	<0.001	0.061	0.012
C18:3 n-6, γ-ALA	1.91	2.27	3.21	4.40	3.12	3.02	9.37	4.59	0.74	0.089	0.438	0.174
C18:3 n-3, α-ALA	84,90	84.36	71.58	63.57	87.04	74.30	90.61	79.87	1.11	<0.001	0.380	0.193
CLAcis9trans11	0,32	0.31	1.08	1.08	0.68	0.72	1.09	1.22	0.24	<0.001	0.731	0.791
C20:2	5,82	6.43	9.52	8.61	6.71	7.58	7.32	8.93	0.55	<0.001	0.518	0.112
C20:4n-6, AA	68,30	49.12	61.53	110.22	86.55	84.78	69.16	94.54	2.60	<0.001	0.609	0.016
C22:2	1,00	1.02	1.19	1.13	1.04	1.45	1.96	1.42	0.32	0.405	0.816	0.451
C20:5n-3, EPA	15,65	5.90	29.16	25.41	14.18	20.05	15.64	19.84	1.07	<0.001	0.528	0.080
C22:4	3,14	3.19	4.60	4.68	1.93	2.58	2.73	3.89	0.47	<0.001	0.247	0.459
C22:5 n-3, DPA	8,11	8.00	14.94	12.66	6.78	10.17	8.79	10.48	0.84	<0.001	0.680	0.180
C22:6 n-3, DHA	25,17	15.55	16.01	10.37	11.57	8.37	17.23	10.40	1.01	<0.001	0.040	0.051
SFA	664,99	894.37	640.87	804.81	588.48	716.22	802.47	885.09	4.29	0.004	0.184	0.165
MUFA	749,69	917.59	734.08	832.79	666.18	697.76	924.20	979.88	4.54	0.021	0.126	0.065
PUFA	712,82	532.27	766.85	850.35	732.94	768.22	810.71	875.59	4.30	<0.001	0.077	0.007
n-6	575,85	415.27	630.56	733.66	611.44	652.75	675.71	751.11	4.11	0.003	0.077	0.007
n-3	136,97	117.00	136.29	116.69	121.50	115.47	135.00	124.48	1.50	<0.001	0.841	0.030
Long Chain PUFA	130,01	102.73	142.18	180.75	133.96	139.57	139.61	157.26	2.30	<0.001	0.077	0.007
n-6/n-3	4,20	3.55	4.63	6.29	5.03	5.65	5.01	6.03	0.47	<0.001	0.086	0.002
HFI	4,09	2.92	3.29	2.99	4.30	3.91	3.82	3.72	0.30	<0.001	0.058	0.055
Δ5,6 desaturase	18,24	17.42	18.54	21.26	18.28	18.17	17.22	17.96	0.97	0.055	0.083	0.094

CB: crossbreed Robusta Maculata x Sasso. LD: Lohmann Dual. NN: Naked neck. RJ: Red JA57. GEN: Genotype. ENR: Enrichment. INT: Interaction GEN x ENR. LA: linoleic acid; α-ALA: α-linolenic acid; AA: arachidonic acid; EPA: eicosapentaenoic acid; DPA: docosapentaenoic acid; DHA: docosahexaenoic acid; SFA: saturated fatty acids; MUFA: monounsaturated fatty acids; PUFA: polyunsaturated fatty acids; HFI: Healthy Fatty Index.

These changes differentially affected the amount of n-3 and n-6 long-chain PUFA which are higher in the muscle of fatter chicken (RJ and NN). In this composite mechanism of gaining resources and in the use and storage of these resources, heavier strains showed a higher storage ability of dietary inputs (Mattioli et al., 2022b). This fact was particularly evident in the breast because the drumstick of kinetic active birds uses long chain PUFA (C > 20), by the way of β-oxidation, for kinetic activity (Failla et al., 2021). Indeed eicosapentaenoic (**EPA**), docosapentaenoic (**DPA**) and DHA acids were higher in the ENR group of chickens with higher grass pecking (RJ, NN and to a lesser extent CB) than LD in both meat cuts (breast *p* > 0.05).

Long chain PUFA in animals are produced starting from several elongation and desaturation steps of relative precursors (linoleic acid, LA n-6; α-linolenic acid, ALA n-3). In this pathway, the limiting factor is retained the Δ6 desaturase, which acts two times during the entire process (Tosi et al., 2014). Several papers assessed that the Δ5,6 desaturase activity of different chicken strains is affected by genetic strain and that this value is higher in SG and local breeds (Boschetti et al., 2016; Castellini et al., 2016). In this experiment, the muscles of CB showed higher Δ5,6 desaturase activity confirming the specificity of some metabolic pathways as already shown for tocol profile.

The HFI was also affected by genetic strain. The rationale of this index was to consider the dietary lipid input from a nutritional viewpoint and its analysis showed that all the chicken strains have a good nutritional lipid composition (Dal Bosco et al., 2022). In that study, two genotypes organically reared, extremely different in growth rate and lipid and fatty acid composition, were compared. The Authors found that HFI of SG chickens showed significantly better values respect to that of FG chickens. In the present experiment, the average HFI value was 3.46 and 3.86, respectively for breast and drumstick, demonstrating their general excellent nutritional quality.

Another consideration is that the enrichment applied improved the index thanks to the reduction of fat and SFA, and the increased PUFA and n-3 series, with a reduction of n-6/n-3 ratio.

## CONCLUSIONS

Our previous research activities in extensive poultry farming, assessed that “*It is useless to a give large space to chickens that are not able to exploit it… but at the same time it is useless to use chickens capable of grazing without providing them with grass*”. In the present trial, the first requirement is certainly satisfied: available space and grass (and even the possibility of hiding or taking shelter) whereas regarding the ability of chicken to move and graze, it possible to state that the four SG genotypes showed strengths and weaknesses.

The combination of environmental (geographical position, photoperiod, soil, climate, plant essences, water, presence of wild animals etc.) and anthropic situations make difficult to give generalized solutions of genetic adaptability. Apart from behavior, performance and meat quality, other factors such as resistance to challenge factors (temperature, microbial pressure), biodiversity, environmental impact, and availability of chicken strains play significant roles in this choice.

**Figure 3:**
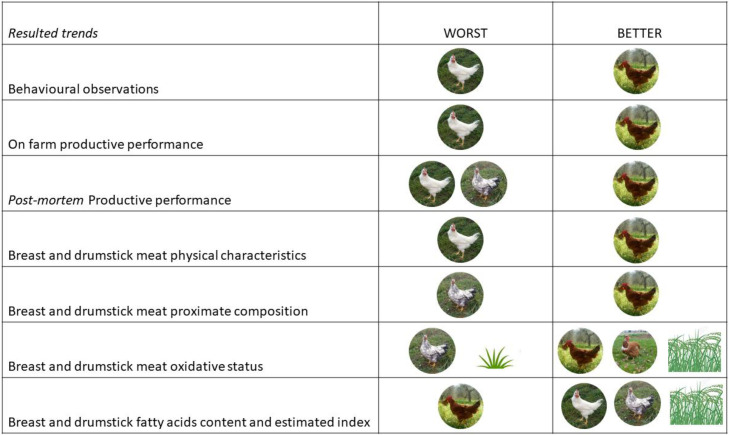
Resulted trend of the macro-groups of variables analysed. The round pictures depict chicken genotypes with a worst or better trend resulted in the different macro-groups of variables analyzed. Trends related to the presence (ENR, bush picture) or absence (NO ENR, small grass picture) of environmental enrichments were also depict.

In this specific case the 4 poultry genotypes showed good adaptation capabilities and sufficient production performance, with the exception of LD and CB, which were too light for the commercial supply chain. Although with a certain variability, the physical-chemical characteristics also appeared to be good; from a nutritional point of view they were excellent in terms of fatty acids profile and content of bioactive molecules (Figure 3).

Further studies regarding the capacity of the chicken genotypes to finalize the walking activity in the foraging, the metabolic destiny of dietary resources and the characteristics of outdoor enrichments should be better defined.

## DISCLOSURES

The authors declare no conflicts of interest.

## Appendix Supplementary materials

Image, application 1

## Footnotes

Supplementary material associated with this article can be found in the online version at doi:10.1016/j.psj.2024.104131.
