# Supplementary material for: Effect of genotype and outdoor enrichment on productive performance and meat quality of slow growing chickens
Source: Poult Sci. 2024 Jul 29;103(10):104131. doi: 10.1016/j.psj.2024.104131 (PMC11350495; doi:10.1016/j.psj.2024.104131)
Supplement: Supplementary file 1 [file mmc1.doc]

Supplementary tables

Table S1. Description of the detected behaviours in chickens outdoor reared.

| **BEHAVIOUR** | **ATOL references^1^** | **DESCRIPTION** |
| --- | --- | --- |
| **Roosting** | ATOL_0000837 | Chicken in a lying position with the ventral body region in contact with the floor. |
| **Resting** | ATOL_0000816 | Chicken with the body in line with the ground, the head is erect and eyes opened. Only the feet are in contact with the floor. |
| **Sleeping** | ATOL_0000873 | Chicken with the head in a low posture (under the wing or on the litter) and eyes closed. |
| **Walking** | ATOL_0000805 | Chicken that moves more than three steps. |
| **Running** | ATOL_0000806 | Chicken that moves more than three steps quickly. |
| **Feed pecking** | ATOL_0000363 | Chicken that pecks inside the feeder. |
| **Drinking** | ATOL_0000361 | Chicken that pecks inside drinker. |
| **Other pecking** | ATOL_0000845 | Pecking other things. |
| **Self-grooming** | ATOL_0000823 | Animal preening its own feathers. |
| **Swelling** | ATOL_0005361 | Chicken puffing out the breast feathers. |
| **Scratching** | ATOL_0000360 | Chicken that scratches with the paw on the ground. |
| **Stretching** | ATOL_0000822 | Chicken that stretches the body and legs. |
| **Wings flapping** | ATOL_0000822 | Chicken that beats its wings with breast protruding and vertically extended posture. |
| **Dust bathing** | ATOL_0000824 | Chicken that forces the sand or other materials into the plumage by squatting on the ground and making appropriate movements with the body, wings and leg. |
| **Allo-grooming** | ATOL_0000826 | Chicken that preens the feathers of another conspecific. |
| **Grass pecking** | ATOL_0000844 | Chicken that pecks the grass. |
| **Attacking** | ATOL_0000813 | Chicken that fighting against any conspecific. |
| **Escaping** |  | Chicken that escapes from another conspecific. |
| **Sheltering** |  | Chicken that moves through the grass and the bushes in order to find a shelter until disappearing. |

^1^ Traits follow the ATOL ontology (<https://www.atol-ontology.com/en/atol-2/>), in accordance with the PILLOW project data management plan.

Table S2. Data obtained in the experimentation

| **Macro-variables** | **Traits analysed** |
| --- | --- |
| Behavioural observations | Walking  Grass pecking  Eating habits |
| On farm productive performance | Chick weight  Slaughtering weight  DWG  DFI  FCR |
| *Post-mortem* Productive performance | Ready to cook carcass weight  Bust yield  Breast weight  Breast yield  Breast thickness  Abdominal fat  Sternum length  Tibia length  Boneless drumstick weight  Bone drumstick weight  Meat/Bone ratio |
| Breast and drumstick meat physical characteristics | pHu  L*  a  b  Drip loss  WHC |
| Breast and drumstick meat proximate composition | Moisture  CP  EE  Ash |
| Breast and drumstick meat oxidative status | Retinol  γ-T3  α-T3  δ-T  γ-T  α-T  Σ Tocols  Thiols  TBARS  Carbonyls  Lipid-protein cross reactive |
| Breast and drumstick fatty acids content and estimated index | C14  C16  C16:1  C17  C17:1  C18  C18:1 n-9  C18:2 n-6. LA  C18:3 n-6. γ-ALA  C18:3 n-3. α-ALA  CLAcis9trans11  C20:2  C20:4 n-6. AA  C22:2  C20:5 n-3. EPA  C22:4  C22:5 n-3. DPA  C22:6 n-3. DHA  SFA  MUFA  PUFA  n-6  n-3  Long Chain PUFA  n-6/n-3  HFI  Δ5,6 desaturase |
